# Investigation of III-Nitride MEMS Pressure Sensor for High-Temperature Applications

**DOI:** 10.3390/mi17020177

**Published:** 2026-01-28

**Authors:** Makhluk Hossain Prio, Maruf Morshed, Lavanya Muthusamy, Md Sohanur E. Hijrat Raju, Itmenon Towfeeq, Durga Gajula, Goutam Koley

**Affiliations:** 1 Holcombe Department of Electrical and Computer Engineering, Clemson University, Clemson, SC 29634, USA; marufm@clemson.edu (M.M.); mraju@clemson.edu (M.S.E.H.R.); gkoley@clemson.edu (G.K.); 2 Texas Instruments, Dallas, TX 75243, USA; lmuthus@clemson.edu; 3 Modjoul Inc., Greenville, SC 29615, USA; itmenon@modjoul.com; 4 Institute for Matters and Systems (IMS), Georgia Institute of Technology, Atlanta, GA 30332, USA; dgajula3@gatech.edu

**Keywords:** COMSOL simulation, gallium nitride, high temperature, MEMS, piezoelectric, polarization, pressure sensor, pressure transducer, sensitivity, strain

## Abstract

High-temperature operation of AlGaN/GaN Heterojunction Field Effect Transistor embedded diaphragm-based MEMS pressure sensors have been investigated, which utilized their wide bandgap and piezo resistivity to perform stably at elevated temperatures. The performance of the pressure sensor was observed over a change in applied pressure of 35 kPa, which resulted in an experimentally measured change in drain–source resistance (Δ*R_DS_/R_DS_*_(0)_) of 0.32% at room temperature and 0.65% at 250 °C, respectively. Additionally, the COMSOL-based Finite Element (FE) Simulations, in conjunction with our developed theoretical model, was utilized to theoretically determine the change in drain–source resistance. This theoretically calculated Δ*R_DS_/R_DS_*_(0)_ of 0.45% at room temperature closely aligns with the experimental observations. Moreover, the sensor exhibited a gate-bias-dependent tunability, with the enhancement of sensitivity under increasingly negative gate voltages. Furthermore, the sensors demonstrated a stable and repeatable sensing operation over multiple pressure cycles up to 300 °C, with a rapid response time of <10 ms, suggesting excellent potential for reliable, high-performance pressure sensing in harsh, high-temperature environments.

## 1. Introduction

Pressure sensors operate by transducing applied pressure into measurable electrical outputs, such as changes in resistance, voltage, or capacitance [1,2]. In harsh environments, such as in the aerospace, automotive, oil and gas, deep-well drilling, nuclear power, and petroleum industries, there is a great need for high-temperature-compatible pressure sensors [3,4,5,6,7,8]. At present, piezoresistive [9], capacitive [10], piezoelectric [11], and triboelectric [12] pressure sensors have been utilized for high-temperature applications [1,2,13,14]. Additionally, fiber optic sensors demonstrate thermal stability in extreme and flammable environments for oil and gas downhole applications due to their characteristics of using optical fiber as the sensing medium [5,6,8].

Recent advancements in microelectromechanical systems (MEMS) technology have enabled substantial progress in the design of pressure sensors and actuators incorporating MEMS-based sensing elements. Applications in sectors such as oil and gas, automotive, defense, aerospace/avionics, and nuclear power increasingly demand MEMS devices capable of reliable operation under harsh environmental conditions, including elevated temperatures, high humidity, mechanical shock and vibration, overload stress, radiation exposure, and corrosive chemical environments [15]. In addition to miniaturization, MEMS pressure sensors offer several key advantages: low power consumption, high sensitivity, reduced mass and volume, precise measurement capabilities in confined spaces, low manufacturing cost, minimal disturbance to the measured system, compatibility with monolithic integration of sensing and processing electronics, and suitability for large-scale batch fabrication [2,15,16].

Owing to the narrow bandgap of silicon (~1.12 eV) [17], conventional silicon-based MEMS are typically limited to operating temperatures below ~125 °C [18], and their performance deteriorates significantly above ~150 °C due to the generation of thermal carriers and high leakage currents [4], which makes it less suitable for harsh environments. Silicon also suffers from insufficient corrosion resistance at elevated temperatures, further constraining its use under prolonged extreme conditions [4]. Therefore, scientists have explored solutions for alternative materials and transduction mechanisms capable of maintaining stable operation in such environments [8,15].

Among these, III-Nitride-based MEMS technologies have garnered particular attention. For example, P. Leclaire demonstrated AlN-based MEMS devices [19]. In recent years, AlGaN/GaN HEMT-based MEMS received special interest [4]. It is chemically inert, mechanically stable, and radiation hardened, which makes it a promising device material for hostile environments. The strong polarization fields at the AlGaN/GaN heterointerface induce a two-dimensional electron gas (2DEG) [4], which is strongly responsive to strain. AlGaN/GaN MEMS-based pressure sensors exhibit high sensitivity since both the 2DEG density and the mobility can be modulated with the strain, while only the carrier mobility gets modulated with the applied strain in silicon, providing comparatively lower values for sensitivity. These characteristics of the AlGaN/GaN interface make them better a better-suited material for sensing applications over silicon [4,8,16,20]. Numerous studies have demonstrated the pronounced strain sensitivity of the 2DEG in AlGaN/GaN heterostructures [21,22,23,24,25,26], enabling its effective use for pressure transduction [27,28,29,30]. Additionally, the wide bandgap of GaN (3.4 eV) facilitates stable device performance at elevated temperatures, positioning AlGaN/GaN HEMT-based MEMS as strong candidates for high-temperature sensing applications [8].

AlGaN/GaN heterostructures are commonly realized using metal–organic vapor phase epitaxy (MOVPE) [31], hydride vapor phase epitaxy (HVPE) [32], and molecular beam epitaxy (MBE) [33] growth techniques, with MOVPE offering a key advantage in providing highly uniform, scalable, and device-quality AlGaN/GaN layers suitable for large-area wafers and industrial fabrication. AlGaN/GaN heterostructures grown by atmospheric-pressure MOVPE on GaN demonstrate that epitaxial quality is governed by precursor chemistry and strain management [31]. Increasing Al incorporation via higher trimethylaluminum (TMA) flow reduces the growth rate and degrades crystalline quality due to parasitic reactions and strain relaxation, whereas lowering the trimethylgallium (TMG) flow enhances Al incorporation while improving structural and optical properties, enabling higher Al-content growth [31]. In contrast, lower Al composition AlGaN layers exhibit superior crystalline quality and suppressed crack formation due to minimized lattice mismatch with GaN, making them particularly favorable for high-quality heterostructures and device applications [31].

Another relevant technology for high-temperature pressure sensors is the piezoresistive Si-on-insulator (SOI), which consists of layers of silicon, silicon dioxide, and silicon, as well as other relevant materials. Using Smart-Cut™ processing, SOI pressure sensors with an active silicon layer as thin as 340 nm have demonstrated operation at temperatures approaching 600 °C [34]. For SOI-type pressure sensors, despite their potential for being operated at higher temperatures, the Si material is susceptible to creep at elevated temperatures, leading to its diminished sensitivity and stability. These limitations restrict their applicability in various high-temperature applications, such as aerospace, automotive, and deep-well drilling [17]. Another technique to sense pressure is capacitive-based, which measures changes in pressure due to changes in the distance between electrodes [35]. High-temperature implementations of capacitive sensors have been realized using alumina (Al_2_O_3_) [36] and silicon carbide (SiC) [37] platforms [8]. However, the motion of the capacitive sensor is constrained to small vertical and horizontal movements [38]. If the vertical displacement is large, the capacitance is not suitable for pressure sensing [4]. Given these constraints, research attention has increasingly shifted toward diaphragm-based GaN pressure sensors. In these systems, diaphragm motion—and thus pressure sensitivity—is governed primarily by the material’s intrinsic yield strength rather than by geometric limitations [4]. Coupled with GaN’s high theoretical temperature tolerance, which may reach up to ~600 °C [4,39], AlGaN/GaN-based diaphragm sensors offer a compelling path forward for robust, high-temperature pressure sensing. Additionally, oxidation of III-Nitride films below 500 °C is minimal, as demonstrated by Suria et al. [40].

Additionally, Silicon Carbide’s (SiC) wider bandgap enables their MEMS pressure sensors to be operated at elevated temperatures. Silicon carbide-based piezo resistive pressure sensors at temperatures of up to 600 °C have been studied [41,42,43]; however, these devices demonstrated low output signals and low-pressure sensitivity values [4]. Furthermore, Chen et al. reviewed advances in various types (piezoresistive, capacitive, piezoelectric, fiberoptic, and friction electric) of high-temperature pressure sensors based on inorganic materials (such as silicon, silicon carbide, sapphire, and ceramics) and high-temperature resistant polymers, with detailed analysis on material properties, sensing mechanisms, and temperature compensation methods [1].

Advantages of GaN over SiC include higher mobilities (especially for GaN heterostructures), higher critical break down fields, lower on-resistances, and higher switching speeds [8]. Despite their considerable potential, GaN-based microelectromechanical systems (MEMS) experienced slow technological maturation for many years, largely due to the challenges associated with fabricating III-nitride epilayers on substrates such as SiC and sapphire, which are difficult to etch. Recent advances in the epitaxial growth of high-quality III-nitride films on Si (111) substrates have significantly mitigated these limitations, enabling rapid expansion of III-nitride MEMS research and development. As a result, a wide range of III-nitride–based MEMS devices—including RF filters, pressure sensors, acoustic sensors, and microcantilevers—has now been successfully demonstrated [44].

There have been various studies on AlGaN/GaN-based pressure sensors for room-temperature sensing applications [28,45]. In this work, we have investigated the pressure-sensing characteristics of our AlGaN/GaN Heterojunction Field Effect Transistor (HFET) integrated diaphragm pressure sensor across temperatures ranging from room temperature to 300 °C. We determined the change in drain–source resistance and sensitivity, both experimentally and theoretically, caused by a change in applied pressure. The theoretical values were calculated using COMSOL 6.0-based FE simulations, coupled with a theoretical model. The devices demonstrate stable operation up to 300 °C, underscoring their promise for high-temperature harsh environment applications.

## 2. Materials and Methods

**Figure 1:**
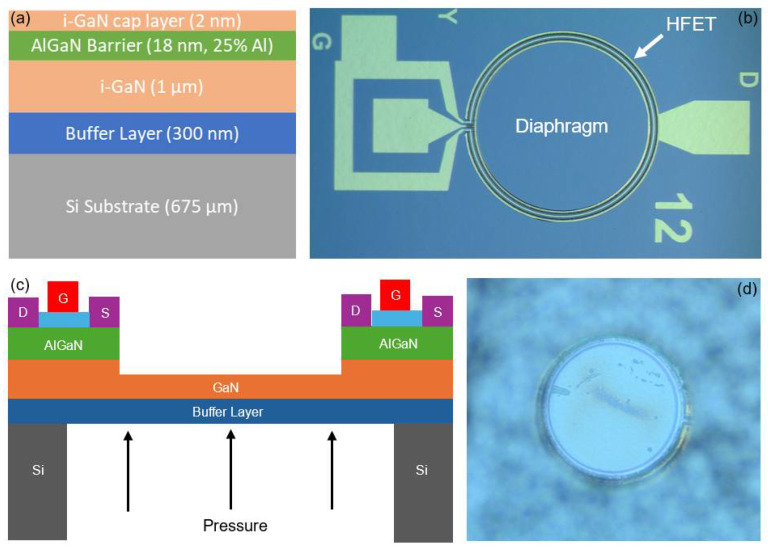
(**a**) Layer structure of the pressure sensor (not drawn to scale); (**b**) optical microscopic image of the diaphragm with integrated HEMT; (**c**) cross-sectional structure of the AlGaN/GaN HFET-embedded pressure sensor; (**d**) optical microscopic image of the backside of the diaphragm, where the pressure was applied.

**Figure 2:**
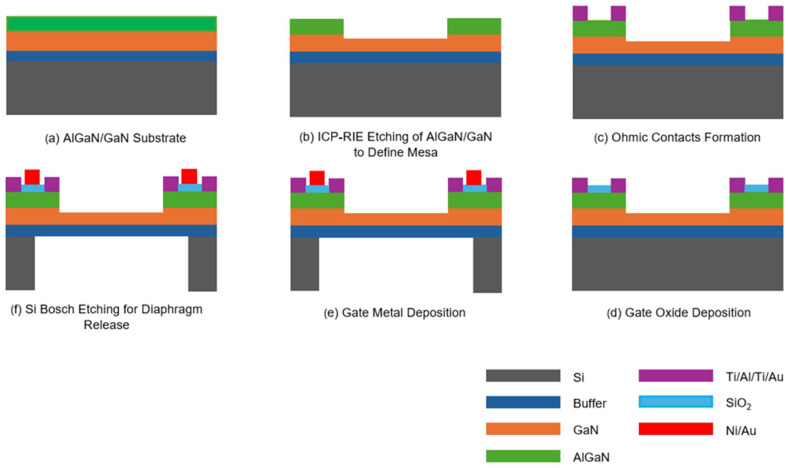
Diagrammatic representation of process flow for the fabrication of pressure sensors. (**a**–**f**) are different steps of the process flow shown in order, as discussed in the text.

The pressure transducers investigated in this study were fabricated on AlGaN/GaN epitaxial layers deposited on (111) silicon wafers, procured from NTT Advanced Technology Corporation, Tokyo, Japan. The wafer structure comprised a 2 nm i-GaN cap layer and an 18 nm Al_0.25_Ga_0.75_N layer atop a 1 µm i-GaN layer, with a 300 nm buffer layer separating the GaN layer from the silicon substrate, which measured 675 µm in thickness. The fabrication process began with etching the uppermost 100 nm of the AlGaN/GaN layer using BCl_3_/Cl_2_ plasma chemistry to define the mesa region around the diaphragm periphery. Subsequently, a metal stack consisting of Ti (20 nm)/Al (100 nm)/Ti (45 nm)/Au (55 nm) was deposited, followed by rapid thermal annealing at 825 °C for one minute to establish ohmic contacts for the source and drain regions of the HFET. Next, a 100 nm layer of SiO_2_ was deposited over the exposed mesa regions using the plasma-enhanced chemical vapor deposition (PECVD) method, serving as the gate dielectric. This step was followed by two sequential metallization stages. The first stage involved depositing a Ni (25 nm)/Au (200 nm) stack as gate metal contacts, and the second stage deposited a Ti (20 nm)/Au (225 nm) stack to form the probe contacts. Finally, the Bosch process was used from the bottom face of the sample to perform through wafer etching of silicon to release the diaphragm. Figure 1a shows the layer structure of the fabricated pressure-sensing device and Figure 1b presents the optical microscopic image of the pressure sensor to reveal its physical structure, with HFET in its periphery connected to the contact pads. The diaphragm has a nominal radius of 500 µm, while the width of the drain, gate, and source is 10 µm each. Moreover, Figure 2 depicts the diagrammatic representation of process flow for the fabrication of pressure sensors.

**Figure 3:**
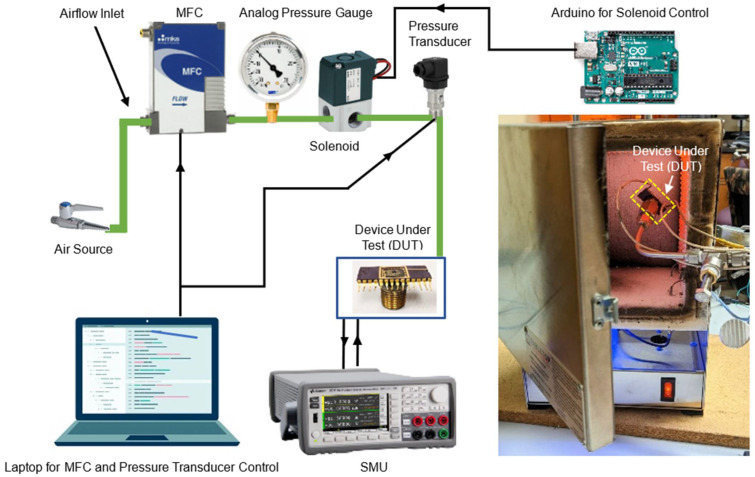
Schematic of experimental setup to investigate the pressure-sensing capability of the AlGaN/GaN Diaphragm-based pressure sensors. The inset shows a photograph of the furnace utilized for high-temperature characterization, with the yellow dashed rectangle indicating the location of the device under test within the furnace chamber.

Controlled air pressures of *P*_1_ and *P*_2_ (*P*_1_ + ∆*P*, where ∆*P* is the change in applied pressure) were maintained at regular intervals utilizing a mass flow controller (MFC) operated through a LabView program and a solenoid valve (Model Name: SMC VT307-5GI-02, SMC Corporation of America, Noblesville, IN, USA), managed by an Arduino Uno R3 microcontroller. To monitor the pressure, an analog pressure gauge (Model Name: WIKA 0–30 in Hg Vacuum/160 PSI, F.N. Cuthbert, Inc., Toledo, OH, USA) was integrated into the air tube line. Additionally, a digital pressure transducer (Model Name: DATAQ 2000361/-HS, DATAQ Instruments, Inc., Akron, OH, USA) was installed, alongside the analog gauge to provide enhanced accuracy in pressure measurements. For testing and characterization, the pressure sensor chip was mounted on a ceramic chip carrier, and the metal contact pads were wire-bonded to the external leads of the carrier. The device was biased at the drain–source terminal using a source measurement unit (Model Name: B2902A Precision Source/Measure Unit, Keysight, Inc., Santa Rosa, CA, USA). For high-temperature testing, the sensor was placed inside a furnace (Model Name: Rapidfire Standard Pro-L, Tabletop Furnace Company, Inc., Lake Katrine, NY, USA) capable of reaching a temperature of 1200 °C. The high-temperature tests up to 300 °C were performed using the tabletop furnace with a ramp rate of 75 °C/min (determined manually). A schematic of the experimental setup is illustrated in Figure 3, showing the MFC, source measurement unit (SMU), pressure gauge, Arduino, and device under test.

## 3. Results and Discussions

### 3.1. Temperature-Dependent I-V Characteristics

**Figure 4:**
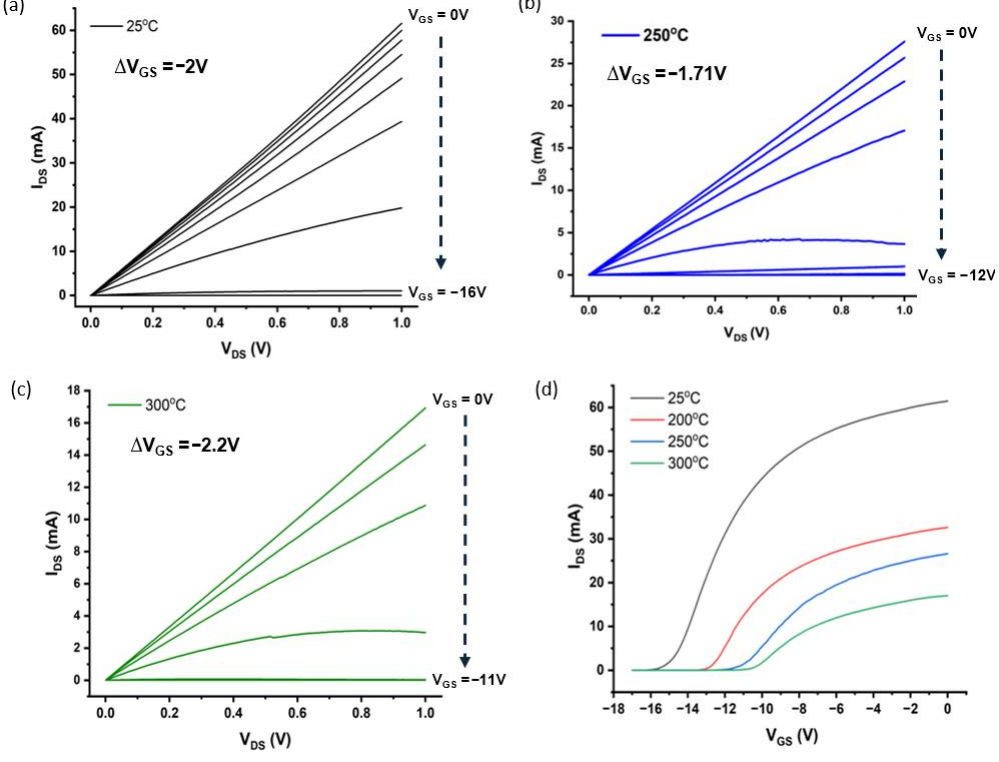
(**a**–**c**) Electrical output characteristics (*I_DS_*-*V_DS_*) of the AlGaN/GaN HFET measured at 25 °C, 250 °C, and 300 °C, respectively. The *V_GS_* decreased from 0 V down to −16 V in increments (ΔV_GS_) of −2 V for (**a**), from 0 V down to −12 V in increments of −1.71 V for (**b**), and from 0 V down to −11 V in increments of −2.2 V for (**c**); (**d**) transfer characteristics (*I_DS_*-*V_GS_*) acquired at multiple temperatures (25 °C, 200 °C, 250 °C, and 300 °C).

A dual-channel source measure unit (SMU) (Keysight B2912A) was used to characterize the *I*–*V* characteristics of the HFET of the device (Device 1) at both room (25 °C) and higher temperatures (200 °C, 250 °C, and 300 °C). In the *I_DS_*-*V_DS_* measurements (see Figure 4a–c), the drain–source current, *I_DS_*, was measured by varying the drain–source voltage (*V_DS_*) from 0 to 1 V, at different constant gate voltages, *V_GS_*. In the *I_DS_*-*V_GS_* measurements (see Figure 4d), while supplying a constant *V_DS_* of 1 V, changes in *I_DS_* due to sweeping the *V_GS_* were measured. the *I_DS_-V_GS_* curves of AlGaN/GaN HFETs with varying temperatures and constant *V_DS_* = 1 V are shown in Figure 4d. A good gate control was observed, as the device shut down at both room and higher temperatures, as presented in Figure 4d. The figure also shows that the absolute value of the gate shutdown voltage decreased (shifted to the right) with the increase in temperature, as the gate shutdown voltage values of −16 V, −13.5 V, −12 V, and −11 V were recorded at 25 °C, 200 °C, 250 °C, and 300 °C, respectively. Additionally, the *I_DS_-V_DS_* curves (Figure 4a–c) reveal a reduction in *I_DS_* for each constant *V_GS_*, as the temperature increased. This decline can be attributed to the decreased carrier mobility, coupled with the reduction in piezo coefficients, at elevated temperatures [46].

### 3.2. Theoretical Modeling for 2DEG Formation and Relationship with Strain

To comprehend the operation of AlGaN/GaN heterostructure-based piezotransistive HFET diaphragms, this section will introduce a theoretical model and elucidate how mechanical deflection of the diaphragm under applied pressure induces variations in the HFET channel resistance.

In the absence of external mechanical strain, the bound polarization sheet charge (*σ_int_*) at the AlGaN/GaN heterostructure interface leads to the formation of a quantum well at the AlGaN/GaN interface, and a two-dimensional electron gas (2DEG) is generated within the well without the need for intentional doping [47,48,49,50,51,52]. Assuming change neutrality through the heterosystem, 2DEG sheet charge concentration at the interfaces is expressed as [51]:

(1) $$n_{s}(x)\;=\;\frac{\sigma_{int}(x)}{q}-\;(\frac{\varepsilon_{o}\varepsilon \left(x\right)}{dq^{2}\;})\;[q\phi_{b}\left(x\right)+E_{F}(x)-∆E_{c}(x)]$$

where *q* is the electron charge, *ε*_0_ is the permittivity of free space, *ε*(*x*) is the relative dielectric constant of AlGaN, *d* is the thickness of the AlGaN layer, *φ_b_* is the Schottky barrier height, *E_F_* is the Fermi level at the heterointerface with respect to the GaN conduction band edge, and Δ*E_c_* is the conduction band offset at the AlGaN/GaN interface. Fermi energy can be calculated using effective electron mass, *m_eff_* = 0.22*m_e_* (where *m_e_* is the electron mass) and reduced Planck’s constant (*ħ*) as *E_F_*(*x*) = *E*_0_(*x*)+ $\frac{\pi \hbar^{2}}{m_{eff}}$ *n_s_*(*x*), where the ground sub-band level of the 2DEG is given by *E*_0_(*x*) = [$\frac{9\pi \hbar q^{2}}{8\varepsilon_{0}\sqrt{8m_{x}^{*}}}$ $\frac{n_{s}(x)}{\varepsilon (x)}$]^2/3^ [47].

**Figure 5:**
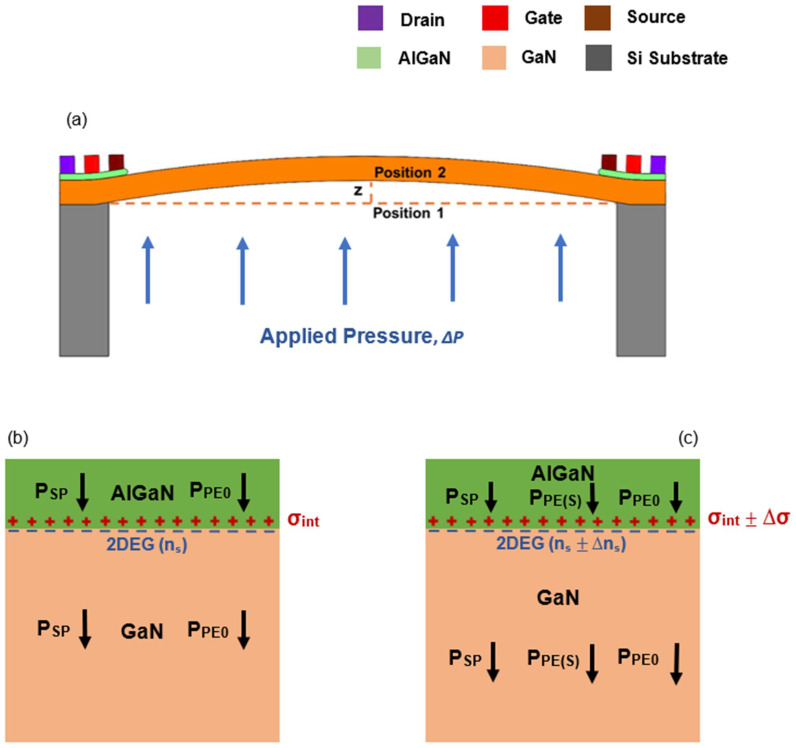
(**a**) Schematic illustration of the AlGaN/GaN HFET-embedded diaphragm-based pressure sensor under an applied pressure, ∆*P*. Spontaneous and piezoelectric polarization at zero applied pressure (**b**), and under an applied pressure, ∆*P* (**c**). Only the piezoelectric component of the polarization PPE(S), induced by the external pressure induced strain, is changing in the AlGaN and GaN layers.

The piezoelectric properties of the AlGaN/GaN heterojunction have been employed to convert diaphragm deflection into an electrical signal in our proposed AlGaN/GaN HFET-embedded diaphragm-based MEMS pressure sensors. This transduction mechanism is illustrated in Figure 5 using a simplified schematic of the diaphragm and the corresponding AlGaN and GaN layers. Figure 5a depicts a schematic of a diaphragm-based AlGaN/GaN pressure-sensing device featuring an HFET located at the diaphragm’s periphery. In the absence of external strain (or pressure), the diaphragm positioned at Position 1 (as shown in Figure 5a) and the corresponding interface charge density (*σ_int_*) can be expressed in terms of spontaneous polarization (*P*_SP_) and piezoelectric polarization (*P_PE_*) as:

*σ_int_* = *P_tot,AlGaN_* − *P_tot,GaN_* = (*P*_*SP*_ + *P*_*PE*0_)*_AlGaN_* − (*P_SP_* + *P*_*PE*0_)*_GaN_* (2)

Figure 5b illustrates the *σ_int_* and corresponding 2DEG density at the AlGaN/GaN interface without external strain.

When the diaphragm is subjected to change in pressure, it deflects to Position 2 (as shown in Figure 5a) by ensuring the displacement from Position 1. External mechanical strain is induced in both the AlGaN and GaN layers, generating additional piezoelectric polarization, denoted as *P_PE_*_(*S*)*AlGaN*_ and *P_PE_*_(*S*)*GaN*_, respectively (shown in Figure 5c), due to the difference in their piezoelectric coefficients. This additional strain induced polarization (Δ*σ*) modifies the 2DEG sheet carrier concentration by Δ*n_s_*. Δ*σ* can be given by the difference in the strain-induced (caused by pressure change) piezoelectric polarization at the interface as [53]:

Δ*σ* = Δ*P*_*PE*(*bending*)_ = *P*_*PE*(*S*) *AlGaN*_ (*x*) − *P*_*PE*(*S*) *GaN*_ = (*e*_31,*AlGaN* −_
*e*_31,*GaN*_)(*ε_x_* + *ε_y_*) + (*e*_33_,*_AlGaN_* − *e*_33,*GaN*_) *ε_z_* (3)

where *x* represents the Al alloy composition in AlGaN, *e*_31_ and *e*_33_ are the piezoelectric constants, and *ε_x_*, *ε_y_*, and *ε_z_* are the average (over the dimensions of the HFET channel) *x*-, *y*-, and *z*-direction strains at the interface due to deflection or bending.

For our proposed pressure sensor, the associated change in 2DEG sheet carrier concentration (Δ*n_s_*) at the AlGaN/GaN heterostructure interface due to Δ*σ* can be given as (considering negligible changes in $\phi_{b}\left(x\right)$, *E_F_*(*x*), and ∆$E_{c}$(x)) [53]:

(4) $$∆n_{s}(x)\;=\;(∆P_{PE(bending)}\;-\;(\frac{\varepsilon_{0}\varepsilon \left(x\right)}{d_{AlGaN}q})\;[q\phi_{b}+E_{F}\left(x)])/[1\;+\;(\frac{\varepsilon \left(x\right)}{\varepsilon \left(0\right)}\right)(\frac{d_{GaN}}{d_{AlGaN}})]\;q$$

where *q* is the electronic charge, $\varepsilon_{o}$ is the permittivity of air, *ε*(*x*) is the dielectric constant of Al_0.25_Ga_0.75_N, *q*$\phi_{b}$ is the Schottky barrier height of the gate contact, *E_F_*(*x*) is the Fermi energy level, and ∆*P_PE_*_(*bending*)_(*x*) is the piezoelectric polarization occurring due to diaphragm bending or displacement.

This change in the sheet carrier concentration (Δ*n_s_*), or the conducting channel carrier density of the HFET, changes the drain–source resistance (*R_DS_*) of the HFET. By maintaining a constant drain–source voltage (*V_DS_*), the change in *R_DS_* (Δ*R_DS_*) can be observed as a corresponding change in the drain–source current (Δ*I_DS_*). Conversely, by maintaining a constant drain–source current (*I_DS_*), the change in *R_DS_* (Δ*R_DS_*) can be observed as a corresponding change in the drain–source voltage (Δ*V_DS_*). This constant drain–source current (*I_DS_*) biasing approach is employed in many practical applications, such as ion-sensitive field effect transistors (ISFETs) [54,55,56] and microcantilever resonance amplitude measurements [57], to compensate for biased point shifts. Hence, any deflection of the diaphragm is transduced into a change in electrical signal (Δ*V_DS_* or ∆*I_DS_*) when a constant *I_DS_* or *V_DS_* is maintained, enabling pressure sensing through the piezotransistive response of the GaN-based diaphragm structure.

### 3.3. Sensor Characterization at Room Temperature

Due to the strong piezoresistive nature of the AlGaN/GaN interface, diaphragm deflection under pressure altered the two-dimensional electron gas (2DEG) density, affecting resistance in our experiment. This change in resistance, $\frac{{∆R}_{DS}}{R_{DS(0)}}$, can be calculated from the relationship among Gauge Factor (GF), 2DEG density, and 2DEG mobility as [4,53,57]:

(5) $$\mathrm{GF}\;=\;\frac{{∆R}_{DS}}{R_{DS(0)}}\times \;\frac{1}{\varepsilon_{av}}=\frac{1}{\varepsilon_{av}}\left(\frac{\Delta \mu_{n}}{\mu_{n}}+\frac{\Delta n_{s}}{n_{s}}\right)$$

Upon simplification, we have:

(6) $$\frac{{∆R}_{DS}}{R_{DS(0)}}(\mathrm{in}\;\mathrm{percentage})=\left(\frac{∆\mu_{n}}{\mu_{n}}+\frac{∆n_{s}}{n_{s}}\right)\;\%$$

where ∆*µ_n_* is the change in mobility, ∆*n_s_* is the change in 2DEG mobility density, *R_DS_*_(0)_ is the initial resistance, ∆*R_DS_* is the change in resistance with average applied strain, and *ε_av_*. *µ_n_* and *n_s_* are the mobility and carrier concentrations, respectively.

Additionally, the Sensitivity (*S*) of the pressure sensor can be determined as [4]:

(7) $$S\;=\;\frac{{∆R}_{DS}}{R_{DS(0)}}\times \;\frac{1}{∆P}\%\;=\;\frac{1}{∆P}(\frac{\Delta \mu_{n}}{\mu_{n}}+\frac{\Delta n_{s}}{n_{s}})\;\%$$

**Figure 6:**
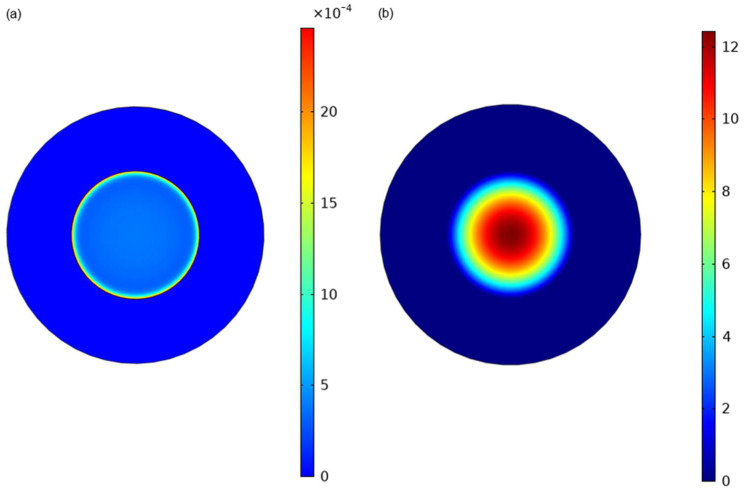
Simulation results for (**a**) strain distribution on AlGaN/GaN Diaphragm using finite element method on COMSOL; (**b**) displacement of the diaphragm with change in applied pressure (35 kPa).

Figure 6 presents Finite Element (FE) simulations conducted using COMSOL Multiphysics (version 6.0, COMSOL Inc., Stockholm, Sweden). Figure 6a illustrates the strain distribution across the diaphragm and Figure 6b shows the diaphragm’s displacement under an applied pressure of 35 kPa above atmospheric pressure. Based on the simulations, the diaphragm’s maximum displacement was approximately 12.2 µm and the displacement is higher at or near the center of the diaphragm. The maximum strain of 24 × 10^−4^ was observed at the circumference of the diaphragm, while the average strain (*ε_av_*) over the dimensions of the HFET channel is 17 × 10^−4^, as illustrated in Figure 6a. Consequently, the HFETs were strategically positioned at the diaphragm’s periphery to maximize the polarization-induced conductivity changes, thereby enhancing device sensitivity. Table 1 shows the Young’s Modulus, Poisson’s Ratio, and density of both GaN and AlGaN considered for the simulation. We note that the vibration noise [58] can influence device behavior. Finite-element vibration simulations in [58] show that the resonant response of AlGaN/GaN diaphragms is primarily governed by tensile residual stress and diaphragm geometry (diameter and thickness), which can lead to a transition between plate-like and membrane-like dynamics that is critical for sensor optimization [58]. In contrast, the simulations and experiments in [59] show that GaN devices exhibit stable responses to humidity variations, which cause negligible effects at elevated temperature (e.g., 360 °C) [59]. We would like to mention here that the impacts of vibration noise and humidity can be effectively minimized by employing a Wheatstone bridge configuration using devices located on and off the diaphragm [60]. This would further enhance the device’s suitability for operation in harsh environments, including aerospace and oil and gas applications.

**Figure 7:**
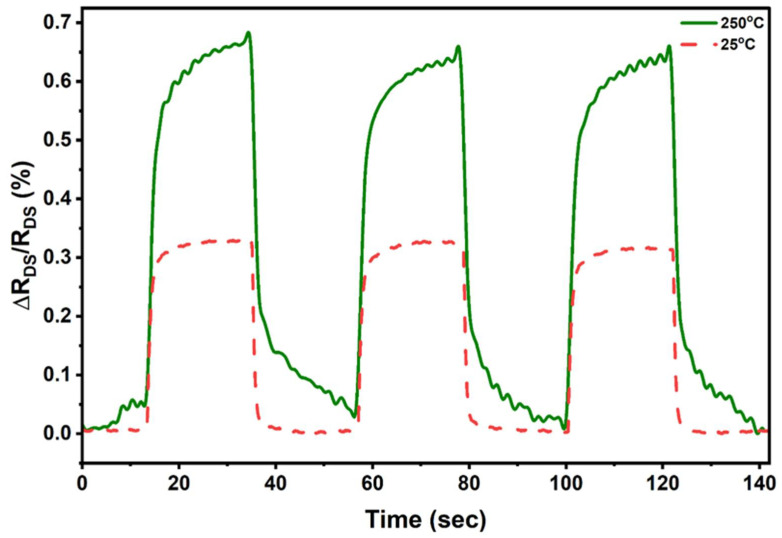
The change in drain–source resistance, measured at room temperature and 250 °C, respectively, under a change in applied pressure of 35 kPa at regular intervals. Measurements were performed for *V_GS_* = 0 V and a constant *V_DS_* = 1 V.

Theoretically, we calculated the values for *n_s_* and ∆*n_s_* as 1.11 × 10^13^ cm^−2^ and −4.6708 × 10^10^ cm^−2^ using Equations (1) and (4), respectively, for our 18 nm thick Al_0.25_Ga_0.75_N layer. The calculated value for *n_s_* is supported by the 2DEG density vs. AlGaN thickness curve in the Supplemental Figure S1. Prior to determining ∆*n_s_*, ∆*P_PE_*_(*bending*)_ was obtained as −5.89 × 10^−9^ cm^−2^ using Equation (3). These values are summarized in Table 2. The fractional change in mobility, due to the change in effective mass, is estimated as Δ*μ_n_/μ_n_* = 0.025% from our prior report [53]. This Δ*μ_n_/μ_n_*, together with the value of Δ*n_s_/n_s_*, yields Δ*R_DS_/R_DS_*_(0)_ = 0.445% at room temperature (RT), following Equation (6). This value of Δ*R_DS_/R_DS_*_(0)_ closely matches the experimentally measured value of Δ*R_DS_/R_DS_*_(0)_ = 0.32% (see Figure 7) at room temperature for Device 1, while exposed to a change in applied pressure of 35 kPa and keeping a constant drain–source voltage, *V_DS_* = 1 V and zero gate voltage. This small mismatch can be attributed to the smaller value in Δ*n_s_* compared to the theoretically calculated value, which can arise from surface and sub-surface carrier trapping—commonly observed in III-nitrides [61] and other wide-bandgap materials that can reduce the effective Δ*n_s_* in our experiments, compared to the theoretical estimates. We note here that the Δ*R_DS_/R_DS_*_(0)_ was obtained with the gate voltage, *V_GS_* = 0 V, which can increase very significantly with more negative gate bias, as discussed below.

**Figure 8:**
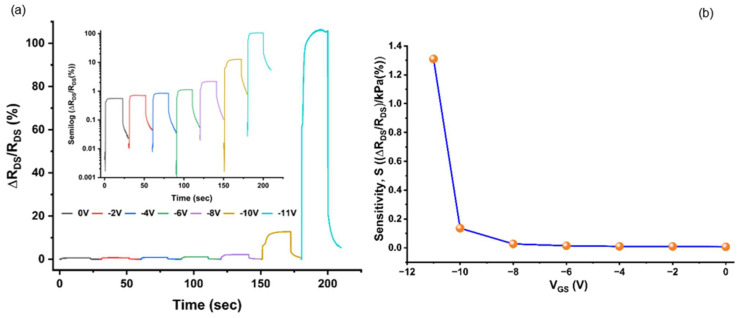
(**a**) The change in drain–source resistance in response to a change in applied pressure of 80 kPa, under different gate biases at room temperature. Inset provides the corresponding semilogarithmic plot; (**b**) sensitivity of the pressure sensor as a function of gate voltage, measured at room temperature.

In our pressure sensors, the sensitivity significantly improves as the gate bias becomes more negative. Figure 8a presents the drain–source resistance changes over time under varying gate voltages for the device 1. At gate biases of −2 V and −8 V, the drain–source resistance (*R_DS_*) changed by 0.7% and 1.1%, respectively, in response to a change in applied pressure of 80 kPa. In comparison, as the gate voltage approached the threshold, under *V_GS_* = −10 V and −11 V, *R_DS_* changed by 11% and 105%, respectively, highlighting the enhanced sensitivity under higher negative gate bias. As shown in Figure 8b, the sensitivity rises dramatically from 0.009%/kPa at *V_GS_* = −2 V to 1.31%/kPa at *V_GS_* = −11 V, representing an approximately 150 times increase. Sensitivity values are calculated using Equation (7). This rise is because the sensitivity S, which is proportional to Δ*n_s_*/*n_s_* (see Equation (7)), increases as *n_s_* reduces due to the application of a more negative *V_GS_* [45]. Incidentally, alongside the increase in sensitivity, the Gauge Factor also increased significantly (150 times) from 4.11 to 617.64, with the increase in negative gate bias from −2 V to −11 V, as determined using Equation (5). High sensitivity and Gauge Factor are particularly desirable in sensors as it simplifies circuit design, reduces the impact of noise, and enhances the resolution of pressure measurements limited by noise [44]. Thus, some of the key advantages of our pressure sensor are its gate bias-related tunability and high deflection sensitivity. Compared with commercial pressure sensor devices, we note that the sensitivity of 1.31%/kPa at higher negative gate bias for our pressure sensor is nearly 90 times higher than the sensors from Omega (Model No: PX409 Series) [62] or PCB Piezoelectronics (Model No: 121A45) [63], which currently have one of the best-performing sensors in the temperature range of <150 °C with a sensitivity of 0.015%/kPa [62,63]. However, more negative gate bias can have the potentially undesirable effect of enhancing charge instability as *R_DS_* increases significantly [64].

**Figure 9:**
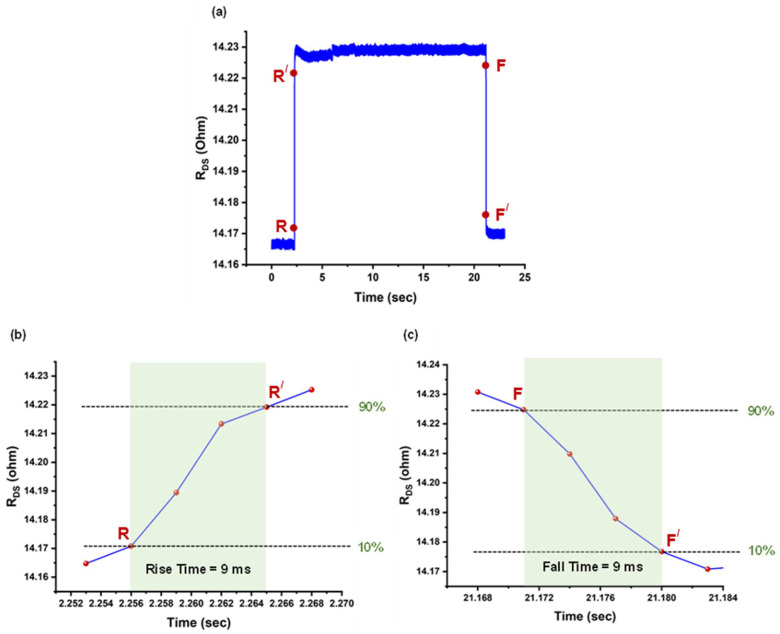
Response time calculation for the pressure sensor, where (**a**) shows the change in resistance with 80 kPa change in applied pressure; (**b**) indicates 9 ms of rise time from R to R/; (**c**) depicts 9 ms of fall time from F to F/.

The response time is a critical parameter for evaluating the performance of a pressure sensor. It is defined as the time taken for a sensor’s output to transition from 10% to 90% of its final value during signal increase, and vice versa, during the signal decay [16]. To determine this parameter for our pressure sensor, the sensor’s response to a change in applied pressure of 35 kPa was recorded, as shown in Figure 9a. Figure 9b shows that upon the introduction of 35 kPa change in applied pressure, the sensor required 9 ms (*τ_rise_*) to reach 90% of its final response from 10%. The transition from 10% to 90% of the final response occurred between 2.256 s and 2.265 s. Similarly, the sensor demonstrated a recovery (decay) time of only 9 ms (*τ_fall_*), as illustrated in Figure 9c, which is considered sufficient for most practical applications [19]. Nevertheless, further improvements in response time may be possible by addressing the sampling rate, limited by the Source Measurement Unit (SMU), and reducing the mechanical flow delay in the tube and the chamber [19]. In fact, the measured rise or fall time includes the response time of the solenoid valve used to perform pressure switching, which can be estimated to be a few milliseconds for 35 kPa pressure change, from the manufacturer datasheet [65].

In addition to the measurements at 35 kPa and 80 kPa applied pressure changes, we evaluated sensor performance at two additional applied change in pressures—30 kPa and 60 kPa—at room temperature and zero gate bias conditions to assess the sensor sensitivity over a broader applied pressure range spanning 30–80 kPa. To perform these experiments, a separate device (Device 2) was used for measurements under same *V_DS_* = 1 V. The device exhibits a good response of Δ*R_DS_/R_DS_*_(0)_ = 0.1% and sensitivity = 0.003%/kPa under a 30 kPa change in pressure, which increased to Δ*R_DS_/R_DS_*_(0)_ = 0.27% and sensitivity = 0.004%/kPa under a 60 kPa applied change in pressure. These results are included in the Supplemental File in Figure S2.

### 3.4. Sensor Characterization at Elevated Temperatures

The pressure-sensing device (Device 1) was tested at 250 °C under identical biasing conditions (*V_DS_* = 1 V, *V_GS_* = 0 V). As shown in Figure 7, the device continues to exhibit a stable response at elevated temperatures, with a measured relative resistance change of Δ*R_DS_/R_DS_*_(0)_ = 0.65% and a corresponding sensitivity of *S* = 0.018%/kPa (determined using Equation (7) under an applied pressure change of 35 kPa), indicating the intrinsic stability and sensitivity of the device performance at higher temperatures. Notably, when the operating temperature increases from RT to 250 °C, the resistance change increases from 0.32% to 0.65% and the sensitivity increases from 0.009%/kPa to 0.018%/kPa. One of the reasons for this increase is that 2DEG is depleted at elevated temperatures [45], coupled with the increase in sheet resistances. Such a decrease in 2DEG density (*n_s_*) can result in an increased sensitivity as Δ*n_s_/n_s_* increases, as can be seen from Equation (7).

**Figure 10:**
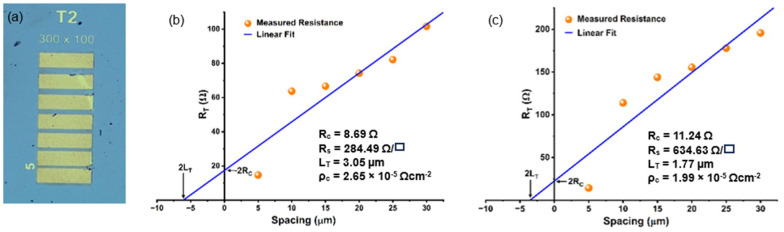
(**a**) Optical microscopic image of the TLM pattern with varying gaps from 5 µm to 30 µm, (**b**,**c**) shows the total resistance vs. spacing curve using the TLM data at 25 °C and 300 °C, respectively.

To verify that increasing temperatures indeed cause 2DEG density and mobility to change, transmission line measurements (TLMs) were carried out at both RT and 300 °C. The TLM structures consisted of probe pads with a width of 100 μm, separated by varying gap lengths ranging from 5 μm to 30 μm. An optical image for TLM pattern is shown in Figure 10a. The resistance values corresponding to each length of semiconductor were measured and plotted as total resistance (*R_T_*) vs. spacing (*l*) for RT and 300 °C, respectively, as shown in Figure 10b,c. After the linear fit of the curves for both RT and 300 °C, the contact resistance (*R_C_*) is calculated from the y-intercept, while the sheet resistance (*R_S_*) is calculated from the slope of the straight line for each. The transfer length (*L_T_*), which signifies the effective length of the contact, can be calculated from the x-intercept. The specific contact resistivity (*ρ*_c_) was calculated as *ρ_c_* = *R_C_ L_T_W* [20].

The measured parameters for TLM patterns are summarized in Table 3. Notably, the contact resistance increased from 8.69 Ω at RT to 11.24 Ω at 300 °C, while the sheet resistance increased nearly 125%, from 284.49 Ω/□ to 634.53 Ω/□ over the same temperature range. This increase in *R_S_* indicates that the higher resistance modulation and enhanced sensitivity observed at elevated temperatures arises from a reduction in 2DEG density, as well as electron mobility. The 2DEG mobility change has been reported to be in the range of 70–80% at elevated temperatures [66], which indicates the rest of the change in sheet resistance can be attributed to the change in 2DEG density.

**Figure 11:**
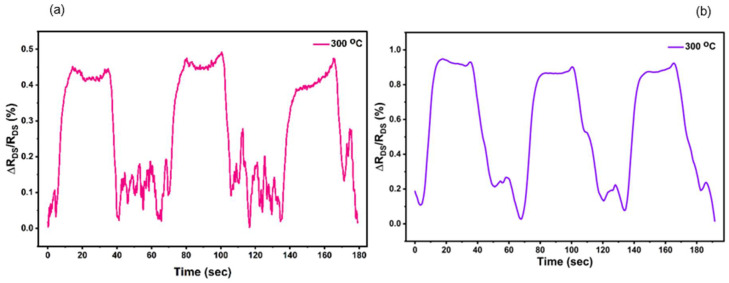
The change in drain–source resistance, measured at 300 °C, under a change in applied pressure of 80 kPa at regular intervals. Measurements were performed for a constant *V_DS_* = 1 V for (**a**) and a constant drain current *I_DS_* = 10 mA for (**b**).

We also investigated the pressure sensor devices at 300 °C. For this, a separate device (Device 3) was utilized to perform similar pressure-sensing experiments at up to 300 °C. However, Device 3 had a smaller diaphragm radius of 375 µm, compared to the 500 µm radius of Device 1. For Device 3, we tested pressure responses at both constant *V_DS_* and constant *I_DS_* conditions. Figure 11a exhibits the pulsed pressure (80 kPa) responses for constant *V_DS_* = 1 V, where the change in drain–source resistance is 0.43% at 300 °C, which is much smaller compared to the 0.65% observed for Device 1 for 35 kPa pressure pulses at 250 °C. The corresponding sensitivities for Device 3 and Device 1 are 0.005%/kPa and 0.018%/kPa, respectively. The magnitude of the pressure pulse was chosen to be higher for Device 3 to account for the reduced strain at the periphery caused by the lower radius of the diaphragm [8]. Since stress (*σ*) is proportional to the square of the diaphragm radius (*r*^2^) [8], we expect the sensitivity of Device 3 to be 1.8 times lower compared to Device 1. However, the experimentally observed sensitivity for Device 3 of 0.005%/kPa was found to be 3.6 times lower compared to Device 1, as discussed above. This variation can be caused by fabrication and material-dependent changes in device behavior.

The pressure sensor performance of Device 3 for constant *I_DS_* = 10 mA (*V_GS_* = 0 V) corresponding to pressure pulses of 80 kPa, for 300 °C, is shown in Figure 11b. Typically, constant *I_DS_* measurements result in more stable sensor response due to the compensation of biasing-related instability [54,55,56,57]. This can be readily seen by comparing the pressure-sensing responses in Figure 11a,b. Clearly, the responses observed in Figure 11b are more stable and less noisy, as expected. Interestingly, the sensitivity is found to be 0.01%/kPa in Figure 11b, which is almost doubled that of the constant voltage case, clearly indicating that constant current-based measurements are preferrable to extracting the best pressure-sensing performance from these devices, especially at higher temperatures, where maintaining device bias stability is critical. Nonetheless, our results from Device 3 clearly underline the potential for the proposed pressure sensors to operate at 300 °C with good stability and reliability.

**Figure 12:**
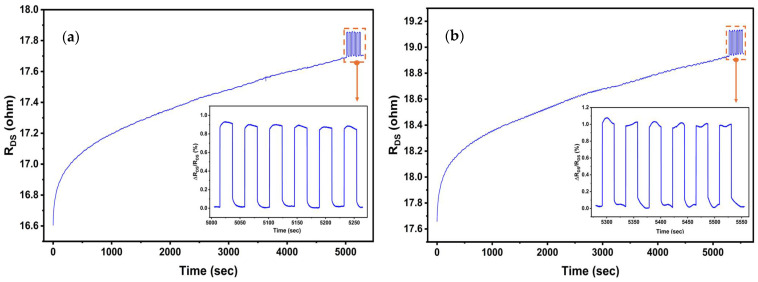
Reliability test of the device under negative gate bias. (**a**) Drain–source resistance variation of the device under −5 V gate bias for around 1.5 h. The insets show magnified plot of change in drain–source resistance with an 80 kPa change in pressure applied; (**b**) *R_DS_* variations and Δ*R_DS_/R_DS_*_(0)_ values were observed for the device under the same *V_GS_* = −5 V gate bias for around 1.5 h, after a two-week interval. All the measurements were performed at a constant *V_DS_* = 1 V.

Furthermore, to test the reliability of devices, especially under the application of a negative gate bias, stress tests were conducted with a gate bias of −5 V applied for over 1.5 h for Device 1. The results are shown in Figure 12. We find that the Device 1 exhibited an increase in drain–source resistance (due to charge trapping related instability), which stayed within ~7%. The pressure-related fractional resistance change (Δ*R_DS_/R_DS_*_(0)_ = 0.9%) was very stable and consistent (inset of Figure 12a) under 80 kPa pressure changes, yielding a sensitivity of 0.011%/kPa. To assess longer-term reliability, the same experiment was repeated after two weeks at *V_GS_* = −5 V (following rigorous testing of various temperatures in between), and the results are shown in Figure 12b. We find the device responses to be very similar, with an initial increase in *R_DS_* under negative gate bias, but stable and consistent fractional drain–source resistance, Δ*R_DS_/R_DS_*_(0)_ = 1%, and sensitivity of 0.012%/kPa, indicating no observable degradation. We would also like to point out that increasing the gate bias to values close to the threshold voltage, i.e., *V_GS_* = −11 V, as in Figure 8, can result in very high-pressure sensitivity, but the device response becomes somewhat unstable due to the much higher degree of charge trapping. Such instability can also be reduced through the use of a Wheatstone bridge [60]; however, for practical applications, an optimal bias point may need to be determined to achieve both high sensitivity and adequate signal stability.

## 4. Conclusions

In summary, we have demonstrated a diaphragm-based AlGaN/GaN HFET-embedded circular membrane pressure transducer, capable of stable and repeatable sensing operation at temperatures of up to 300 °C. The device was characterized under a 35 kPa pressure range, and the experimentally measured resistance change (Δ*R_DS_/R_DS_*_(0)_) showed agreement with theoretical predictions, validating the sensor’s design and modeling accuracy. Additionally, the transducer exhibited gate-bias-dependent tunability, with sensitivity from 0.009%/kPa to 1.31%/kPa, as gate voltage changed from −2 V to −11 V. The device performance was compared for constant voltage and constant current mode at 300 °C, with the latter exhibiting a much more stable response, with almost double the sensitivity. Overall, the diaphragm-based sensor, with the integrated AlGaN/GaN HFET-based deflection transducer, was found to be promising for high-temperature and high-pressure measurements with good stability.

## Figures and Tables

**Table 1:** Parameters for Simulation.

Simulation Parameter (Unit)	GaN	AlGaN
Young’s Modulus (GPa)	265	285
Poisson’s Ratio	0.183	0.3225
Density (kg/m^3^)	6095	3000

**Table 2:** Parameter values for ∆*n_s_*/*n_s_* measurement at RT.

Parameter	Calculated Value
2DEG Density (*n_s_*)	1.11 × 10^13^ cm^−2^
Change in 2DEG Density (∆*n_s_*)	−4.6708 × 10^10^ cm^−2^
∆*P_PE_*_(*bending*)_	−5.89 × 10^−9^ cm^−2^

**Table 3:** Parameter values for TLM Measurement of Device 3 at RT and 300 °C.

Simulation Parameter (Unit)	RT	300 °C
Contact Resistance, *R_c_*	8.69 Ω	11.24 Ω
Sheet Resistance, *R_S_*	284.49 Ω/□	634.63 Ω/□
Transfer Length, *L_T_*	3.05 µm	1.77 µm
Specific Contact Resistivity, *ρ_c_*	2.65 × 10^−5^ Ωcm^−2^	1.99 × 10^−5^ Ωcm^−2^

## Data Availability

The original contributions presented in this study are included in the article. Further inquiries can be directed to the corresponding author.

## Supplementary Materials

The following supporting information can be downloaded at: https://www.mdpi.com/article/10.3390/mi17020177/s1, Figure S1: Plot of 2DEG density vs. AlGaN thickness, showing 18 nm AlGaN thickness yields *n_s_* = 1.1 × 10^13^ cm^−2^; Figure S2: Change in drain–source resistance for 30 kPa and 60 kPa changes in pressure, measured at room temperature over 3 cycles. Measurements were performed at *V_GS_* = 0 V and *V_DS_* = 1 V.
