# Supplementary material for: Investigation of III-Nitride MEMS Pressure Sensor for High-Temperature Applications
Source: Micromachines (Basel). 2026 Jan 28;17(2):177. doi: 10.3390/mi17020177 (PMC12943334; doi:10.3390/mi17020177)
Supplement: Supplementary file 1 [file micromachines-17-00177-s001.zip › micromachines-4035440-supplementary.pdf]

Supplemental Figures

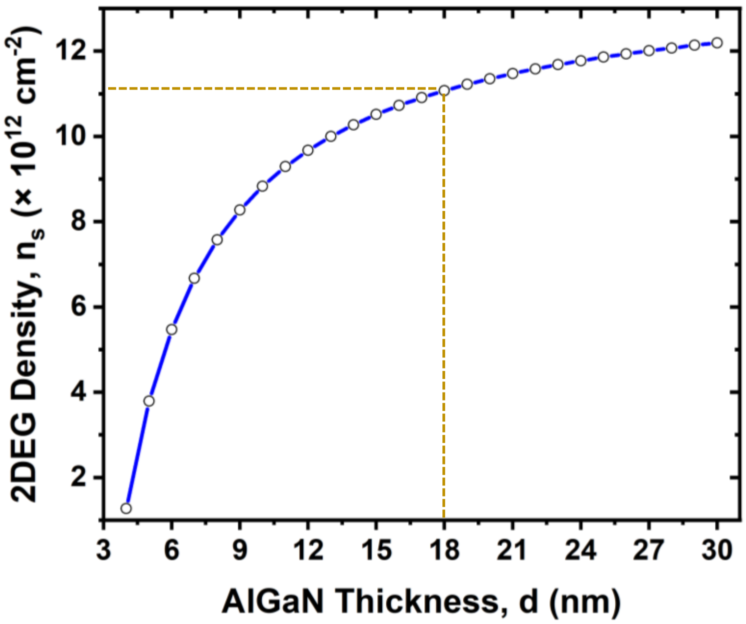

Figure S1. Plot of 2DEG density vs. AlGaN thickness, showing 18 nm AlGaN thickness yields $n_s = 1.1 \times 10^{13}$ cm^{-2}

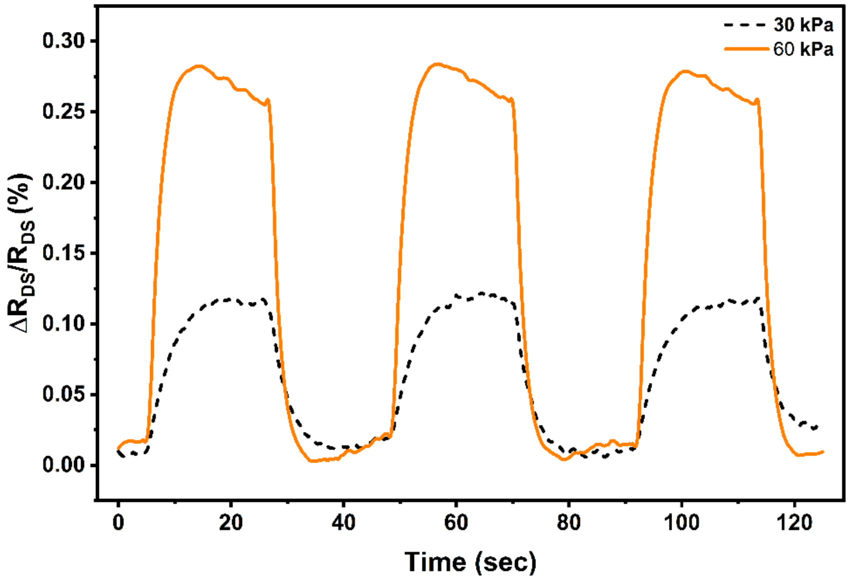

Figure S2. Change in drain–source resistance for 30 kPa and 60 kPa changes in pressure, measured at room temperature over 3 cycles. Measurements were performed at $V_{GS} = 0$ V and $V_{DS} = 1$ V.
